# Latent profiles of professional identity and sleep quality and associations with anxiety symptoms among emergency nurses

**DOI:** 10.3389/fpubh.2026.1806210

**Published:** 2026-03-19

**Authors:** Jingyan Liao, Kun Zhang, Jianna Zhang, Nan Xie

**Affiliations:** 1 Department of Emergency Medicine, West China Hospital, Sichuan University/West China School of Nursing, Sichuan University, Chengdu, China; 2 Disaster Medical Center, Sichuan University, Chengdu, China; 3 Nursing Key Laboratory of Sichuan Province, Sichuan University, Chengdu, China

**Keywords:** anxiety symptoms, emergency nurses, latent profile analysis, professional identity, sleep quality

## Abstract

**Background:**

Emergency nurses are exposed to sustained occupational stress and are at high risk for anxiety symptoms. Professional identity and sleep quality are both closely related to mental health; however, most existing studies have adopted variable-centered approaches that may obscure meaningful heterogeneity within this population.

**Methods:**

A cross-sectional survey was conducted among registered emergency nurses at a tertiary teaching hospital in China between March and June 2024. Participants completed questionnaires assessing professional identity (Professional Identity Scale for Nurses), sleep quality (Pittsburgh Sleep Quality Index), and anxiety symptoms (Generalized Anxiety Disorder-7). Latent profile analysis was performed using the total professional identity score and seven sleep quality component scores as continuous indicators. Differences in anxiety symptoms and demographic characteristics across profiles were examined using chi-square tests and one-way ANOVA.

**Results:**

Three latent profiles were identified: an Adaptive and Well-Functioning profile, a Sleep-Disturbed with Moderate Identity profile, and an At-Risk with Low Identity and Poor Sleep profile. These profiles showed significantly different prevalences of anxiety symptoms, whereas no significant differences were observed in demographic or occupational characteristics across profiles.

**Conclusions:**

Emergency nurses exhibit distinct and clinically meaningful patterns of professional identity and sleep quality that are differentially associated with anxiety symptoms. The identification of a prominent Sleep-Disturbed profile as an important factor associated with higher levels of anxiety symptoms, even among nurses with relatively preserved professional identity. By explicitly capturing heterogeneity that would not be apparent using variable-centered approaches alone, a person-centered perspective may provide insights for the development of more tailored mental health support strategies for emergency nursing populations.

## Introduction

Emergency departments (EDs) are widely recognized as highly demanding healthcare settings, marked by unpredictable workloads, frequent exposure to critical incidents, and persistent time pressures. Nurses working in EDs are consequently particularly susceptible to psychological distress, with anxiety symptoms being highly prevalent (1, 2). Chronic anxiety can compromise nurses' mental well-being, diminish job performance, elevate the risk of burnout, and threaten workforce stability, ultimately impacting the quality and safety of patient care (3, 4). Therefore, identifying modifiable psychosocial and behavioral factors associated with anxiety among emergency nurses represents an important public health priority (2).

Professional identity, an individual's internalized sense of belonging, values, and commitment to the nursing profession, has increasingly been recognized as a crucial psychological resource. A strong professional identity has been linked to greater resilience, higher job satisfaction, and improved mental health outcomes, whereas a weakened or conflicted professional identity may render nurses more susceptible to occupational stress and anxiety (5, 6). Sleep quality is another essential determinant of mental well-being. Irregular shift schedules, night duties, and high cognitive and emotional demands often disrupt sleep among emergency nurses, and such sleep disturbances have consistently been associated with elevated anxiety symptoms (7–9).

Most existing research in this area has employed variable-centered approaches, examining average associations among professional identity, sleep quality, and anxiety (10, 11). Although these studies offer valuable insights, they implicitly assume population homogeneity and may obscure meaningful heterogeneity within emergency nursing populations. In practice, nurses may exhibit distinct patterns of psychological and physiological characteristics. For example, some may maintain a relatively strong professional identity despite substantial sleep disturbances, whereas others may experience concurrent deficits in both areas. Such qualitatively distinct subgroups cannot be easily detected using variable-centered methods alone (12).

Latent profile analysis (LPA) provides a person-centered analytic approach that allows for the identification of unobserved subgroups based on individuals' response patterns across multiple indicators (10, 12). By shifting the focus from average associations to patterns of co-occurring characteristics, LPA can uncover clinically meaningful profiles with potentially different implications for mental health risk and intervention strategies (11, 13). However, person-centered research examining professional identity and sleep quality among emergency nurses remains scarce.

Accordingly, the present study employed LPA to identify latent profiles of professional identity and sleep quality among emergency nurses and to examine the associations of these profiles with anxiety symptoms. By adopting a person-centered perspective, this study seeks to build on existing variable-centered findings and provide a more nuanced understanding of heterogeneity in anxiety-related risk patterns within emergency nursing populations.

## Methods

### Study design and participants

This cross-sectional study was conducted among registered nurses employed in the emergency department of a tertiary teaching hospital in China between March and June 2024. Participants were recruited using a convenience sampling approach. Inclusion criteria included holding a valid nursing license, having at least 1 year of emergency department work experience, and providing voluntary informed consent. Nurses who were interns or students, on extended leave, or reported a history of major psychiatric disorders or diagnosed sleep disorders were excluded.

### Measures

Demographic and occupational characteristics included age, gender, marital status, educational level, professional title, child status, and years of working experience.

Professional identity was assessed using the Professional Identity Scale for Nurses (PISN), which consists of 30 items rated on a 5-point Likert scale (14). Total scores range from 30 to 150, with higher scores indicating stronger professional identity. In the present study, the internal consistency of the PISN was excellent (Cronbach's α = 0.938).

Sleep quality was measured using the Pittsburgh Sleep Quality Index (PSQI), which assesses sleep quality over the previous month across seven components (15). Global scores range from 0 to 21, with higher scores indicating poorer sleep quality. A global PSQI score of 7 or higher was used to define poor sleep quality. The PSQI demonstrated good internal consistency in this sample (Cronbach's α = 0.821).

Anxiety symptoms were assessed using the Generalized Anxiety Disorder-7 (GAD-7) scale (16). Total scores range from 0 to 21, with higher scores indicating more severe anxiety symptoms. Consistent with prior research, a cutoff score of 5 or higher was used to indicate the presence of anxiety symptoms. The GAD-7 showed excellent internal consistency in the present study (Cronbach's α = 0.915).

### Statistical analysis

Statistical analyses were conducted using SPSS version 26.0 and Mplus version 8.3. Descriptive statistics were presented as means and standard deviations for continuous variables and as frequencies and percentages for categorical variables.

Latent profile analysis was performed using the total PISN score and the seven PSQI component scores as continuous indicators. The PSQI component scores were modeled separately rather than using only the global PSQI score to preserve the multidimensional structure of sleep quality. Because latent profile analysis aims to identify distinct patterns across multiple indicators, retaining the individual sleep components (e.g., sleep latency, sleep duration, sleep efficiency, sleep disturbances, and daytime dysfunction) allows for the detection of heterogeneous configurations of sleep characteristics that may not be captured by a single summary score. Models specifying one to five latent profiles were estimated using robust maximum likelihood estimation. Model selection was guided by multiple criteria, including the Akaike Information Criterion (AIC), Bayesian Information Criterion (BIC), sample-size–adjusted BIC (aBIC), entropy, the Lo-Mendell-Rubin adjusted likelihood ratio test (LMR-LRT), profile size, and theoretical interpretability. Although minor residual correlations are common in applied latent profile models, no large residual associations were observed that would indicate meaningful violations of conditional independence.

After selecting the optimal model, participants were assigned to latent profiles based on their most likely posterior membership probabilities for descriptive and comparative analyses. Differences in demographic and occupational characteristics across profiles were evaluated using chi-square tests or one-way analysis of variance, as appropriate. Differences in anxiety symptoms across profiles were examined using chi-square tests, treating anxiety as a binary variable (GAD-7 ≥ 5). The cutoff score of 5 corresponds to established clinical thresholds for at least mild anxiety and is commonly used in epidemiological studies. This dichotomous classification was adopted to facilitate comparison of clinically meaningful anxiety prevalence across latent profiles. When overall differences were significant, *post hoc* pairwise comparisons with Bonferroni adjustment were conducted. A two-tailed *p* value of <0.05 was considered statistically significant. Missing data were minimal across study variables. Descriptive and comparative analyses were conducted using available cases, and latent profile models were estimated using maximum likelihood procedures in Mplus.

### Ethical considerations

This study was approved by the Ethics Committee of West China Hospital of Sichuan University (No. 2023/177). Prior to data collection, written informed consent was obtained from all participants. The study was conducted in accordance with the Declaration of Helsinki, and all data were anonymized to ensure participant confidentiality.

## Results

### Sample characteristics

A total of 179 emergency nurses were included in the final analysis. As presented in Table 1, most participants were female (89.9%), aged 18 to 45 years (93.3%), and married (66.5%). Most participants held a bachelor's degree or higher (89.4%) and had more than 11 years of experience in emergency care (97.2%). The mean professional identity score was 110.91 (SD = 18.46), and the mean global PSQI score was 9.12 (SD = 3.78). Based on established cutoff values, 73.2% of participants reported poor sleep quality (PSQI ≥ 7), and 41.9% reported anxiety symptoms (GAD-7 ≥ 5).

**Table 1:** Demographic and occupational characteristics of participants (*N* = 179).

**Variable**	***N* (%)/Mean ±SD**
**Gender**	
Female	161 (89.9%)
Male	18 (10.1%)
**Age group (years)**	
18–45	167 (93.3%)
46–69	12 (6.7%)
**Education level**	
College	19 (10.6%)
Bachelor	157 (87.7%)
Master or above	3 (1.7%)
**Professional title**	
Junior or low	104 (58.1%)
Intermediate	62 (34.6%)
Senior	13 (7.3%)
**Marital status**	
Married	119 (66.5%)
Single	60 (33.5%)
**Child status**	
Has children	95 (53.1%)
No children	84 (46.9%)
**Years of work experience**	
<5 years	1 (0.6%)
6–10 years	4 (2.2%)
>11 years	174 (97.2%)
Professional identity (PISN)	110.91 ± 18.46
Sleep quality (PSQI total)	9.12 ± 3.78
Poor sleep (PSQI ≥ 7)	131 (73.2%)
Anxiety (GAD-7 ≥ 5)	75 (41.9%)

### Latent profile analysis and model selection

Latent profile analysis was conducted using the total score of the Professional Identity Scale for Nurses and the seven component scores of the Pittsburgh Sleep Quality Index as continuous indicators. Models specifying one to five latent profiles were estimated. Model fit indices are presented in Table 2.

**Table 2:** Model fit indices for latent profile analysis (*N* = 179).

**Number of profiles**	**AIC**	**BIC**	**aBIC**	**Entropy**	**LMR-LRT*(p)***	**Smallest profile (%)**
1	5,412.31	5,458.77	5,420.12	–	–	–
2	5,321.45	5,394.67	5,335.89	0.812	0.002	32.4%
3	5,267.33	5,376.32	5,288.41	0.834	0.021	27.9%
4	5,251.88	5,378.63	5,279.60	0.819	0.058	15.1%
5	5,240.12	5,393.64	5,274.48	0.821	0.124	11.2%

The three-profile solution was selected as the optimal model based on lower AIC/BIC/aBIC, higher entropy, significant LMR-LRT, and theoretical interpretability.

Although information criteria (AIC, BIC, and aBIC) continued to decrease with increasing numbers of profiles, model selection was based on a combination of statistical indices and substantive considerations. The three-profile solution demonstrated a favorable balance between statistical fit and parsimony, with relatively low information criterion values, acceptable entropy (0.834), a significant Lo–Mendell–Rubin adjusted likelihood ratio test (*p* = 0.021), and adequate profile sizes. An entropy value above 0.80 is generally considered indicative of good classification quality, suggesting satisfactory separation among the identified profiles.

While the four-profile solution showed marginally lower AIC and BIC values, the LMR-LRT was not statistically significant (*p* = 0.058), suggesting that the four-profile model did not provide a significantly better fit than the three-profile model. In addition, the additional class identified in the four-profile solution was a comparatively smaller class and did not exhibit clinically distinct characteristics beyond those observed in the three-profile solution.

Therefore, taking into account statistical evidence, parsimony, classification accuracy, and interpretability, the three-profile model was retained as the optimal solution.

### Description of latent profiles

The three latent profiles were characterized based on the conditional means of professional identity and PSQI component scores (Table 3).

**Table 3:** Characteristics of the three latent profiles (*N* = 179).

**Variable**	**Adaptive (*n* = 50)**	**Sleep-disturbed (*n* = 79)**	**At-risk (*n* = 50)**	***F*/χ^2^**	***p***
Professional identity (PISN)	135.6 ± 12.4	115.8 ± 10.2	88.7 ±15.3	125.43	<0.001
PSQI total	4.2 ± 1.5	8.9 ± 1.8	12.1 ± 3.0	189.76	<0.001
Sleep latency	0.8 ± 0.6	1.9 ± 0.7	2.5 ± 0.8	85.34	<0.001
Sleep duration	0.9 ± 0.7	1.6 ± 0.8	2.2 ± 0.9	42.12	<0.001
Sleep efficiency	0.5 ± 0.5	1.2 ± 0.6	1.8 ± 0.7	67.89	<0.001
Sleep disturbances	1.0 ± 0.4	1.6 ± 0.5	2.1 ± 0.6	58.23	<0.001
Daytime dysfunction	0.5 ± 0.5	1.8 ± 0.7	2.5 ± 0.8	112.45	<0.001
Anxiety (GAD-7 ≥ 5), *n* (%)	4 (8.0%)	31 (39.2%)	40 (80.0%)	68.91^*^	<0.001

Values are presented as mean ± SD or *n* (%). χ^2^ tests were used for categorical variables. *Post hoc* Bonferroni tests indicated significant differences between all profiles (*p* < 0.05).

^*^χ^2^ test for categorical variable.

### Adaptive and well-functioning profile (*n* = 50)

This profile was characterized by the highest professional identity scores and the lowest scores across all PSQI components, indicating strong professional identity and good overall sleep quality. Participants in this group reported short sleep latency, adequate sleep duration, high sleep efficiency, minimal sleep disturbances, and low levels of daytime dysfunction.

### Sleep-disturbed with moderate identity profile (*n* = 79)

Participants in this profile exhibited moderate professional identity scores together with elevated PSQI component scores, particularly in sleep latency and daytime dysfunction. Overall sleep quality was moderately impaired, suggesting notable sleep-related difficulties despite relatively preserved professional identity.

### At-risk with low identity and poor sleep profile (*n* = 50)

This profile was characterized by the lowest professional identity scores and the highest PSQI component scores. Participants reported prolonged sleep latency, reduced sleep duration, poor sleep efficiency, frequent sleep disturbances, and pronounced daytime dysfunction, reflecting concurrent deficits in professional identity and sleep quality.

### Differences in anxiety symptoms across latent profiles

Significant differences in anxiety symptoms were observed across the three latent profiles (Table 3). The prevalence of anxiety symptoms (GAD-7 ≥ 5) was lowest in the Adaptive profile (8.0%), intermediate in the Sleep-Disturbed profile (39.2%), and highest in the At-Risk profile (80.0%). Between-profile differences were statistically significant (χ^2^ = 68.91, *p* < 0.001). *Post hoc* pairwise comparisons with Bonferroni correction indicated that all three profiles differed significantly from one another in anxiety prevalence (all adjusted *p* < 0.05).

### Demographic and occupational characteristics across latent profiles

Comparisons of demographic and occupational variables across the three latent profiles are presented in Table 4. No significant differences were observed across profiles with respect to gender, age group, educational level, marital status, child status, professional title, or years of working experience (all *p* > 0.05). These findings indicate that latent profile membership was primarily differentiated by professional identity and sleep quality rather than by demographic or occupational characteristics.

**Table 4:** Comparison of demographic and occupational variables across latent profiles (*N* = 179).

**Variable**	**Adaptive (*n* = 50)**	**Sleep-disturbed (*n* = 79)**	**At-risk (*n* = 50)**	***F*/χ^2^**	***p***
Gender (Female)	45 (90.0%)	73 (92.4%)	45 (90.0%)	0.32	0.852
Age (18–45 years)	48 (96.0%)	74 (93.7%)	47 (94.0%)	0.25	0.882
Bachelor degree or above	46 (92.0%)	72 (91.1%)	46 (92.0%)	0.04	0.980
Married	34 (68%)	52 (65.8%)	33 (66.0%)	0.06	0.970
Has children	33 (66.0%)	52 (65.8%)	33 (66.0%)	0.00	1.000
Work experience >11 years	47 (94.0%)	74 (93.7%)	46 (92.0%)	0.18	0.915
Professional title (Intermediate/Senior)	20 (40.0%)	29 (36.7%)	19 (38.0%)	0.16	0.924

No significant differences were observed across latent profiles (all *p* > 0.05).

As a supplementary variable-centered analysis, nurses with anxiety symptoms reported significantly lower professional identity and poorer sleep quality than those without anxiety symptoms (Table 5).

**Table 5:** Comparisons of professional identity and sleep quality between nurses with and without anxiety symptoms (*N* = 179).

**Variable**	**No anxiety (*n* = 104)**	**Anxiety (*n* = 75)**	**Test statistic**	***p***	**Effect size**
Professional identity (PISN) (*M* ± SD)	119.7 ± 19.5	104.7 ± 20.4	*t* = 4.99	<0.001	*d* = 0.75
PSQI total scores (*M* ± SD)	7.4 ± 3.2	10.1 ±3.9	*t* = −5.07	<0.001	*d* = 0.76
Poor sleep (PSQI ≥ 7), *n* (%)	58 (55.8%)	61(81.3%)	χ^2^ = 13.65	<0.001	ϕ = 0.28

Values are presented as mean ± SD or *n* (%). Independent-samples *t* tests were used for continuous variables, and χ^2^ tests for categorical variables.

## Discussion

This study employed a person-centered latent profile analysis to identify distinct patterns of professional identity and sleep quality among emergency nurses and to examine their associations with anxiety symptoms (17). Three clinically meaningful profiles were identified: an Adaptive and Well-Functioning profile, a Sleep-Disturbed with Moderate Identity profile, and an At-Risk profile characterized by Low Identity and Poor Sleep. These profiles differed markedly in the prevalence of anxiety symptoms, whereas no significant differences were observed in demographic or occupational characteristics (18).

The most notable finding was the identification of a prominent Sleep-Disturbed with Moderate Identity profile, which comprised a substantial proportion of the sample. Nurses in this profile exhibited moderately preserved professional identity alongside marked difficulties with sleep initiation and daytime functioning (19). Importantly, the prevalence of anxiety symptoms in this group was substantially higher than that observed in the Adaptive profile, suggesting that sleep disturbance is strongly associated with elevated anxiety symptoms even when professional identity is relatively intact. This pattern underscores that sleep quality may represent an important correlate of anxiety symptoms, rather than serving solely as an intermediary correlate of professional identity.

The At-Risk profile was characterized by the co-occurrence of low professional identity and poor sleep quality and was associated with the highest prevalence of anxiety symptoms (20). This pattern is conceptually consistent with the conservation of resources (COR) theory, which proposes that simultaneous deficits across psychological and physiological domains may increase vulnerability to psychological distress (21). In this context, professional identity may be understood as a psychological resource reflecting meaning, role coherence, and professional commitment, whereas adequate sleep represents a fundamental physiological resource necessary for emotional regulation and cognitive functioning. The simultaneous impairment of both domains may therefore reflect cumulative resource loss, which COR theory suggests can intensify psychological strain. In contrast, the Adaptive profile represented a favorable configuration in which strong professional identity coexisted with good sleep quality, a combination potentially linked to greater emotional resilience (22).

These person-centered findings complement existing variable-centered research demonstrating associations among professional identity, sleep quality, and anxiety (5). Rather than assuming population homogeneity, the present analysis shows that these factors cluster into distinct configurations within the emergency nursing workforce (19). For example, the Sleep-Disturbed profile indicates that interventions focusing solely on enhancing professional identity may be insufficient for a substantial subgroup if sleep problems are not addressed concurrently (23). Conversely, the At-Risk profile may necessitate more comprehensive strategies that target multiple domains of vulnerability simultaneously (6).

From a practical perspective, these findings support the development of profile-specific mental health strategies. Although causal conclusions cannot be drawn, the observed associations suggest that nurses in the At-Risk profile may potentially benefit from integrated interventions targeting sleep disturbances, professional identity, and anxiety symptoms, potentially including cognitive-behavioral therapy for insomnia, mentoring, and psychological support (24). For nurses in the Sleep-Disturbed with Moderate Identity profile, preventive interventions focusing on sleep health—such as sleep education tailored to shift work and organizational initiatives to promote adequate rest—may be particularly effective. In contrast, nurses in the Adaptive profile may primarily require maintenance-oriented strategies aimed at preserving existing protective resources (25).

The use of a single-center convenience sample may limit the generalizability of the findings. The sample was relatively homogeneous, particularly in terms of years of work experience, with a large proportion of participants reporting more than 11 years of emergency nursing experience. This restricted variability may have reduced the ability to detect potential demographic or occupational differences across latent profiles. Moreover, institutional culture, workload characteristics, and features of the regional healthcare system in China may differ from those in other contexts, which may further limit the transferability of the findings to broader emergency nursing populations. Future multi-center studies including more diverse and heterogeneous samples are needed to strengthen external validity.

In addition, anxiety was analyzed as a dichotomous variable using an established clinical cutoff. While this approach facilitates interpretation in terms of symptom prevalence and clinical relevance, it may have reduced variability and statistical power compared with analyses based on continuous anxiety scores.

Despite these limitations, this study highlights the added value of a person-centered approach for understanding heterogeneity in the mental health of emergency nurses. The identification of a distinct Sleep-Disturbed profile emphasizes that clinically relevant risk patterns may not be evident from variable-centered analyses alone. By explicitly capturing these configurations, latent profile analysis can inform more precise and responsive mental health interventions for high-risk nursing populations and further support the translation of these findings into practice. Importantly, given the cross-sectional design, the present findings reflect associations rather than causal relationships. It remains unclear whether sleep disturbance and professional identity contribute to anxiety symptoms, whether anxiety influences these factors, or whether bidirectional relationships exist. Longitudinal studies are needed to clarify temporal and causal pathways.

## Conclusion

In conclusion, this study identified three distinct and clinically meaningful latent profiles of professional identity and sleep quality among emergency nurses using a person-centered analytic approach. These profiles differed markedly in the prevalence of anxiety symptoms, highlighting heterogeneity in anxiety-related risk patterns within a population that is often treated as homogeneous.

Notably, the identification of a prominent Sleep-Disturbed with Moderate Identity profile underscores the strong association between sleep disturbance and anxiety symptoms, even among nurses whose professional identity remains relatively intact. This finding suggests that sleep problems warrant focused attention as a core target for mental health prevention and support, rather than being regarded solely as a secondary correlate of psychological resources.

By explicitly capturing distinct configurations of professional identity and sleep quality that would not be evident using variable-centered approaches alone, this study demonstrates the added value of a person-centered perspective for understanding anxiety risk among emergency nurses. These findings may guide the development of more precise, profile-tailored mental health strategies that align interventions with the specific needs of different subgroups within the emergency nursing workforce.

## Data Availability

The raw data supporting the conclusions of this article will be made available by the authors, without undue reservation.
